# Supplementary material for: Prevalence and severity of secondary traumatic stress and optimism in Indian health care professionals during COVID-19 lockdown
Source: PLoS One. 2021 Sep 28;16(9):e0257429. doi: 10.1371/journal.pone.0257429 (PMC8478227; doi:10.1371/journal.pone.0257429)
Supplement: Attachment — Submitted filename: PONE-D-21-14477_reviewer.pdf [file pone.0257429.s002.pdf]

PLOS ONE

Prevalence and severity of secondary traumatic stress and optimism in healthcare professionals in India during COVID-19 lockdown.

--Manuscript Draft--

**Manuscript Number:**	PONE-D-21-14477
**Article Type:**	Research Article
**Full Title:**	Prevalence and severity of secondary traumatic stress and optimism in healthcare professionals in India during COVID-19 lockdown.
**Short Title:**	Secondary traumatic stress and optimism in Indian healthcare professionals during COVID-19 lockdown.
**Corresponding Author:**	Neha Parashar, PhD<br>Maastricht University Faculty of Psychology and Neuroscience: Universiteit Maastricht Faculty of Psychology and Neuroscience<br>Maastricht, NETHERLANDS
**Keywords:**	COVID-19, Secondary traumatic stress, Healthcare professionals, Mental health, Optimism
**Abstract:**	Background: The COVID-19 pandemic has brought to light the lacunae in the preparedness of healthcare systems across the globe. This preparedness also includes the safety of healthcare providers (HCPs) of various levels. Sudden widespread of COVID-19 infection has created threatening and vulnerable conditions for the HCPs. The current pandemic situation has not only affected physical health of HCPs but also their mental health.<br>Objective: This study aims to understand the prevalence and severity of secondary traumatic stress, optimism parameters, along with states of mood experienced by the HCPs, viz., doctors, nurses and other medical professionals, during the COVID-19 lockdown in India.<br>Methodology: The assessment of level of secondary traumatic stress (STS), optimism/pessimism (via Life Orientation Test-Revised) and current mood states experienced by Indian HCPs in the present COVID-19 pandemic situation was done using a primary data of 2008 HCPs from India during the first lockdown during April-May 2020. Data was collected through snow-ball sampling technique, reaching out to various medical health care professionals through social media platforms.<br>Result: Obtained results have shown that among HCPs, 77%(n=1548) of doctors experienced severe to high levels of secondary traumatic stress as compared to other HCPs, which includes nurses and other medical professionals i.e., 23%(n=460), who were reported little to no secondary traumatic stress. Female HCP's showed higher levels of STS and were also found to be higher on the optimism as compared to male in the sample.<br>Conclusion: The current investigation has revealed that there is a dire need of creating better and accessible mental health programmes and facilities for the health care providers in India.
**Order of Authors:**	Manohar KN
	Neha Parashar, PhD
	CR Satish Kumar
	Sanjiv Rao
	Sekhar Y
	Vijay Kumar K
	Amalselvam A
	Hemkumar TR
	Prem kumar BN
	Sridhar K

Powered by Editorial Manager® and ProduXion Manager® from Aries Systems Corporation

	Pradeep Kumar S
	Sangeeta K
	Shivam .
	Chetan Kumar
	Vivek Verma
	Judith .
**Additional Information:**	
**Question**	**Response**
**Financial Disclosure**<br><br>Enter a financial disclosure statement that describes the sources of funding for the work included in this submission. Review the submission guidelines for detailed requirements. View published research articles from *PLOS ONE* for specific examples.<br><br>This statement is required for submission and **will appear in the published article** if the submission is accepted. Please make sure it is accurate.<br><br>**Unfunded studies**<br>Enter: *The author(s) received no specific funding for this work.*<br><br>**Funded studies**<br>Enter a statement with the following details:<br>• Initials of the authors who received each award<br>• Grant numbers awarded to each author<br>• The full name of each funder<br>• URL of each funder website<br>• Did the sponsors or funders play any role in the study design, data collection and analysis, decision to publish, or preparation of the manuscript?<br>• **NO** - Include this sentence at the end of your statement: *The funders had no role in study design, data collection and analysis, decision to publish, or preparation of the manuscript.*<br>• **YES** - Specify the role(s) played.<br><br>* typeset	The author(s) received no specific funding for this work.
**Competing Interests**<br><br>Use the instructions below to enter a	The authors have declared that no competing interests exist.

competing interest statement for this submission. On behalf of all authors, disclose any competing interests that could be perceived to bias this work—acknowledging all financial support and any other relevant financial or non-financial competing interests.<br><br>This statement is required for submission and **will appear in the published article** if the submission is accepted. Please make sure it is accurate and that any funding sources listed in your Funding Information later in the submission form are also declared in your Financial Disclosure statement.<br><br>View published research articles from *PLOS ONE* for specific examples.<br><br>**NO authors have competing interests**<br><br>Enter: *The authors have declared that no competing interests exist.*<br><br>**Authors with competing interests**<br><br>Enter competing interest details beginning with this statement:<br><br>*I have read the journal's policy and the authors of this manuscript have the following competing interests: [insert competing interests here]*<br><br>* typeset	
**Ethics Statement**<br><br>Enter an ethics statement for this submission. This statement is required if the study involved:<br><br>• Human participants<br>• Human specimens or tissue<br>• Vertebrate animals or cephalopods<br>• Vertebrate embryos or tissues<br>• Field research	The approval was obtained from The Ethics committee of Manipal hospitals, Bangalore (ECR/34/Inst/KA/2013/RR-19). Our investigation process remained anonymous, and no identifiers (such as name, address, email id, phone numbers, name of hospital employed) were collected. Every participant was informed about and understood the purpose of our investigation before entering the study.

Powered by Editorial Manager® and ProduXion Manager® from Aries Systems Corporation

Write "N/A" if the submission does not require an ethics statement.

General guidance is provided below. Consult the submission guidelines for detailed instructions. **Make sure that all information entered here is included in the Methods section of the manuscript.**

Format for specific study types

Human Subject Research (involving human participants and/or tissue)

- Give the name of the institutional review board or ethics committee that approved the study
- Include the approval number and/or a statement indicating approval of this research
- Indicate the form of consent obtained (written/oral) or the reason that consent was not obtained (e.g. the data were analyzed anonymously)

Animal Research (involving vertebrate animals, embryos or tissues)

- Provide the name of the Institutional Animal Care and Use Committee (IACUC) or other relevant ethics board that reviewed the study protocol, and indicate whether they approved this research or granted a formal waiver of ethical approval
- Include an approval number if one was obtained
- If the study involved *non-human primates*, add *additional details* about animal welfare and steps taken to ameliorate suffering
- If anesthesia, euthanasia, or any kind of animal sacrifice is part of the study, include briefly which substances and/or methods were applied

Field Research

Include the following details if this study involves the collection of plant, animal, or other materials from a natural setting:

- Field permit number
- Name of the institution or relevant body that granted permission

**Data Availability**<br><br>Authors are required to make all data underlying the findings described fully available, without restriction, and from the time of publication. PLOS allows rare exceptions to address legal and ethical concerns. See the PLOS Data Policy and FAQ for detailed information.<br><br>A Data Availability Statement describing where the data can be found is required at submission. Your answers to this question constitute the Data Availability Statement and **will be published in the article**, if accepted.<br><br>**Important:** Stating 'data available on request from the author' is not sufficient. If your data are only available upon request, select 'No' for the first question and explain your exceptional situation in the text box.<br><br>Do the authors confirm that all data underlying the findings described in their manuscript are fully available without restriction?	No - some restrictions will apply
**Describe where the data may be found in full sentences. If you are copying our sample text, replace any instances of XXX with the appropriate details.**<br><br>• If the data are **held or will be held in a public repository**, include URLs, accession numbers or DOIs. If this information will only be available after acceptance, indicate this by ticking the box below. For example: *All XXX files are available from the XXX database (accession number(s) XXX, XXX.*).<br>• If the data are all contained **within the manuscript and/or Supporting Information files**, enter the following: *All relevant data are within the manuscript and its Supporting Information files.*<br>• If neither of these applies but you are able to provide **details of access elsewhere**, with or without limitations, please do so. For example:	The data could be available on requesting the Principal Investigator (Dr KN Manohar; email: drmanohar_kn@yahoo.com).

Data cannot be shared publicly because of [XXX]. Data are available from the XXX Institutional Data Access / Ethics Committee (contact via XXX) for researchers who meet the criteria for access to confidential data.<br><br>*The data underlying the results presented in the study are available from (include the name of the third party and contact information or URL).*<br>• This text is appropriate if the data are owned by a third party and authors do not have permission to share the data.<br><br>* typeset	
Additional data availability information:	

Powered by Editorial Manager® and ProduXion Manager® from Aries Systems Corporation

Manuscript

Click here to access/download;Manuscript;0 Manuscript Final 01.05.2021.docx

Manohar KN[1], Neha Parashar[2], C.R. Satish Kumar[3], Sanjiv Rao[4], Sekhar Y[5], Vijay Kumar K[6], Amalselvam A[7], HemkumarTR[8], Premkumar BN[9], Sridhar K[10], Pradeep Kumar S[11], Sangeeta K[12], Shivam[13], Chetan Kumar[14], Vivek Verma[15]and Judith[16]

[1]Consultant Physician & Diabetologist - Manipal Hospital, Bengaluru, India; Email: drmanohar_kn@yahoo.com

[2]PhD student, University of Maastricht (Netherlands); Clinical Psychologist; Bengaluru, India; Email: n.parashar@maastrichtuniversity.nl

[3]PhD student, University of Maastricht, Consultant Clinical Psychologist Manipal Hospital; Bengaluru, India; Email: Satish.kumar@maastrichtuniversity.nl

[4]Consultant, Internal Medicine- Manipal Hospital;Bengaluru, India; Email:drsanjivrao@gmail.com

[5]Consultant, General Physician- Manipal Hospital; Bengaluru, India; Email: yadsekhar06@gmail.com

[6]Diabetologist, Narayana Medical Centre, Bengaluru, India, Email:vijayk.kris@gmail.com

[7]General Physician; Joseph Medical Centre, Bengaluru, India, Email:dramal.selvam@yahoo.com

[8]General Physician, Internal Medicine, Sakra World Hospital; Bengaluru, India; Email: trhemkumar@gmail.com

[9]Physiotherapist; Kempegowda Institute of Medical Sciences, Bengaluru, India; Email:premkumarbn@gmail.com

[10]General Physician, Diabetologist, Sharada Medical Center, Bengaluru, India; Email: diabdoc99@gmail.com

[11]Anaesthesiologist, Fortis Hospital, Bengaluru, India; Email: spradeep_kumar@yahoo.com

[12] Physician, Consultant, Manipal Hospital, Bengaluru, India; Email: drsangeeta321@gmail.com

[13]General Physician, Sharada Medical Centre, Bengaluru, India; Email: dr.shivammamc@gmail.com

[14]Medicine Intern, Vydehi Medical College, Bengaluru, India; Email: chethan.k.b1996@gmail.com

[15] Department of Statistics, Assam University, Silchar, Assam, India; Department of Neurology, All India Institute of Medical Sciences (AIIMS), New Delhi, India; Email: viv_verma456@yahoo.com

[16]Nursing Superintendent, Manipal Hospitals, Bengaluru, India; Email: judith.vasan@manipalhospitals.com

Prevalence and severity of secondary traumatic stress and optimism in health care professionals in Indian during COVID-19 lockdown.

Abstract

Background: The COVID-19 pandemic has brought to light the lacunae in the preparedness of healthcare systems across the globe. This preparedness also includes the safety of healthcare providers (HCPs) of various levels. Sudden widespread of COVID-19 infection has created threatening and vulnerable conditions for the HCPs. The current pandemic situation has not only affected physical health of HCPs but also their mental health.

Objective: This study aims to understand the prevalence and severity of secondary traumatic stress, optimism parameters, along with states of mood experienced by the HCPs, *viz.*, doctors, nurses and other medical professionals, during the COVID-19 lockdown in India.

Methodology: The assessment of level of secondary traumatic stress (STS), optimism/pessimism (via Life Orientation Test-Revised) and current mood states experienced by Indian HCPs in the present COVID-19 pandemic situation was done using a primary data of 2008 HCPs from India during the first lockdown during April-May 2020. Data was collected through snow-ball sampling technique, reaching out to various medical health care professionals through social media platforms.

Result: Obtained results have shown that among HCPs, 77%(n=1548) of doctors experienced severe to high levels of secondary traumatic stress as compared to other HCPs, which includes nurses and other medical professionals i.e., 23%(n=460), who were reported little to no secondary traumatic stress. Female HCP’s showed higher levels of STS and were also found to be higher on the optimism as compared to male in the sample.

Conclusion: The current investigation has revealed that there is a dire need of creating better and accessible mental health programmes and facilities for the health care providers in India.

Keywords: COVID-19, Secondary traumatic stress, Healthcare professionals, Mental health, Optimism

1. Introduction

Mental health risks have always been a concern in health care professionals, but the sudden outbreak of the COVID-19 pandemic brought forward the hidden lacunae in preparation for risk management in the health care community. Patients come to health care providers with physical illness, but the psychological burden and trauma of the illness also dealt with during the treatment gets translated into secondary/vicarious trauma of the health care provider.[1,2]

Recent studies on Chinese health care professionals who dealt with the first and largest outbreak of the COVID-19 had shown that the frontline health care workers such as doctors and nurses faced depressive symptoms, insomnia and anxiety as compared to non-frontline healthcare workers. [3,4,5] The ~~following~~ findings are also supported by a meta-analysis and systematic review done by Pappa and colleagues which indicated the prevalence of anxiety, insomnia and depression in the primary health care professionals.[6] Similar results were also found in a large scale survey done on Indian health care professionals where they found that the health care professionals reported higher rates of burnouts post COVID era. [7, 8]

Secondary traumatic stress (STS), being one of the primary concern at hand, is a group of symptoms including intrusion, avoidance, and arousal. [9] These STS symptoms share similarities with those of posttraumatic stress disorder (*PTSD*), as suggested in the 4th edition of the Diagnostic and Statistical Manual of Psychiatric Disorders [DSM-IV; APA].[10] However, unlike PTSD, STS could be due to indirect contact in a professional context (e.g., caring for a traumatized patient). [11] Dealing with the COVID-19 affected patients; the roles of HCPs evolved drastically, now venturing into areas other than their area of specialization due to the lack of professionals available to keep up with the demand; this has created occupational stress as a cause for worry among them [12,13,14, 15,16].

HCPs with perceived risk and fear of getting COVID-19 indicated positively predicted depression, anxiety, and stress [16]. Reports suggest that in addition to work stress, HCPs had faced new problems such as being verbally and physically assaulted by a mob [17]. A shortage in personal protective equipment (PPE) was also a significant concern among healthcare professionals [18]. Furthermore, improper training and guidelines regarding PPE raised several concerns [19]. One of the major protective factors among healthcare professionals was their optimistic attitude to cope with the stress related to their profession.

Optimism defined as the ability to look at the brighter side of things. Optimism helps health care workers experience improved life satisfaction alongside improved outcomes with their patients. Specific skills such as optimism, interpersonal skills, hope, and faith can protect one's mental health. In a sense, optimism could be used as a coping mechanism to ensure the mental health of HCPs and can help them be more resilient in difficult times. Resources such as psycho-social support, leisure time and improvement in infrastructure adaptations in hospitals could help improve their mental health [17].

Lack of literature on exploring the mental health status and possible coping factors of the Indian health care professionals during pandemic leaded to the current study's development. Hence, the present study aims to report secondary traumatic stress levels, optimism, and mood states experienced by the HCPs within the Indian subcontinent during the COVID-19 pandemic.

2. Material and Method

Participants and procedures

For the present cross sectional study, information associated with the level of secondary traumatic stress (STS), optimism and mood state during COVID-19 among health care professionals in India was collected. The Google forms were circulated on various groups and social media (LinkedIn, WhatsApp) and invited health care professionals from different cities across India to complete the questionnaire voluntarily. Due to lockdown and to reduce human contact and transmission risk related with the disease; online platforms such as Google forms and social media were utilized as a mode of data collection. The data collection took place between the months of April 2020-May 2020, which was the first national lockdown for first wave of COVID-19 to hit India.

Additionally, we also sent the questionnaire to many health care professionals who had cooperated with us, and used their contact network to spread the questionnaire, utilizing snowballing method. For the safeguarding of data (also mentioned in ethical clearance document); all the data was collected from primary research supervisor’s institutional email address and every two weeks the data was removed and secured in an external hard drive which was not connected with internet and was not accessible to anyone but the primary researchers.

The respondents were English proficient health care professionals above 20 and below 65 years of age in India (including doctors, nurses, physiotherapist, dentist, lab technicians and post graduate medical students). Before collecting responses, in the consent form and safeguard process for maintaining the anonymity of the data; we stated the purpose of the investigation, and responses were collected only after obtaining the consent. This questionnaire was anonymous. The survey began on April 16, 2020, and ended on May 15, 2020, when India was in a complete lockdown period due to outbreak of COVID-19. Due to the nature of Google forms any incomplete questionnaires were not accepted. We collected a total of 2153 questionnaire out of which only 2008 were valid and finally used for analysis. We excluded those observations which were inconsistent or were inappropriately filled and those which were not consented. Across India the data was collected from 23 states and one Union Territory (viz., Karnataka, West Bengal, Tamil Nadu, Maharashtra, Gujarat, Uttar Pradesh, Andhra Pradesh, Telangana, Kerala, Delhi, Haryana, Assam, Tripura Jharkhand, Odisha, Bihar, Uttarakhand, Punjab, Rajasthan, Chhattisgarh, Goa, Manipur, Andaman and Nicobar Islands, and Madhya Pradesh).

3. Measurements

In order to understand the socio-demographic profile of the population, information on individual's age, gender, marital status, occupation (doctors, nurses, others), years of experience, type of practice (clinic and hospital) and their current state of practice were collected. The mental health status was assessed using below describes scales:

I. Secondary Traumatic Stress Scale

The Secondary Traumatic Stress Scale (STS) is a self-report inventory designed to assess the frequency of STS symptoms in professional caregivers. As used in the current manuscript, STS refers to Post Traumatic Stress Disorder symptoms and diagnoses that arise from exposure to the traumatic events of others.

The STSS is a 17-item measure explicitly designed to assess the effects of healthcare providers' exposure to secondary trauma from patient experiences [11]. Unlike other measures that include items related to burnout or compassion satisfaction, the 17 STSS items correspond to the 17 DSM-IV PTSD symptoms for Criteria B (Intrusion), C (Avoidance), and D (Arousal; American Psychiatric Association). [10] Respondents indicate how often they

experienced each symptom in the past seven days on a Likert-type scale ranging from 1 ("never") to 5 ("very often"). In place of assessing Criterion A of the diagnostic guidelines for PT± (trauma exposure): the STSS uses "prompts" that suit professional's setting, for instance. In order to fit the emergency room environment, the word "client" was changed to "patient" in all relevant items. By replacing Criterion A (trauma exposure) for DSM-IV PTSD with these prompts, the STSS largely mirrors PT± from a secondary stressor, which is the definition of STS used in the present study. The fact that the STSS closely mirrors the DSM-IV criteria for PTSD allows it to be validly compared—albeit with some caution—to DSM-IV PTSD [20, 21].

STSS score grouped into five categories based on the total score. STSS score below 28 corresponds to "little or no STS," score between 28 and 37 means "mild STS," between 38 and 43 "moderate STS," between 44 and 48 "high STS," and beyond 49 "severe STS". In prior research, the STS showed good psychometric properties. The STSS has acceptable psychometrics as measured by convergent (mean $r = 0.39$) and discriminant (mean absolute $r = 0.07$) validity. The STSS has high overall internal consistency based on Cronbach's alpha values ($\alpha = 0.93$), and acceptable internal consistency for the symptom cluster sub-scales (Intrusion, $\alpha = 0.80$; Avoidance, $\alpha = 0.87$; Arousal, $\alpha = 0.83$). Additionally, its tree- structure model is supported by confirmatory factorial analysis, although the factors are inter-correlated . [10]

II. Life Orientation Test-Revised

Life orientation test revised (LOT-R) is a 10-item scale that measures how optimistic or pessimistic people feel about their future. Respondents use a 5-point rating scale (0 = strongly disagree; 4 = strongly agree) to show how much they agree with 10 statements about positive and negative expectations. These statements include "In uncertain times, I usually expect the best" and "If something can go wrong for me, it will." The internal consistency (Cronbach's alpha) ranged between .74 and .78 [22].

III. Mood (Visual Analogue Scale)

Mood visual analogue scale (VAS) (0- extremely sad to 10- extremely happy) is a psychometric response scale which is used to measure subjective characteristics or attitudes and have been used in the past for measuring the multitude of disorders [23].

4. Statistical analysis

To estimate the prevalence and severity of secondary traumatic stress, optimism parameters, along with states of mood experienced by the HCPs, viz., doctors, nurses and other medical professionals based on their socio-economic characteristics viz., age, experience in field, secondary traumatic stress symptoms (*intrusion, avoidance and arousal)*, mood visual analogue scale, marital status, clinical role, type of practice were described with the help of mean, standard deviation and percentages, respectively, were collected. For the exploratory analysis, mean and standard deviations of continuous variables and proportions for categorical variables were reported to describe the levels of secondary traumatic stress (STS), optimism/ pessimism, and current mood states in the sample based on profession and gender of the healthcare professionals in the study. Regression analysis was further used to explore changes in secondary traumatic stress, optimism and mood states. Significance of all statistical tests' were defined as bilateral $P<0.01$. SAS university edition was used to analyse data in the study.

5. Results

General characteristics

The number of participants who participated was 2153, of which complete information was available for 2008 (93%) individuals, which is considered as a population for present study. Among completers, 1027(51.15%) were females. Mean age was 35.7(± 11.9) years; females (mean 29.7 [± 8.9] years) were younger than males (mean 41.9 [± 11.5] years). The majority were married (60.2%), percentage of married males (80.2%) was twice of the females. Most HCPs were nurses (924, 46%) followed by doctors (611, 30.4%) and remaining belongs to other professional groups. The population is classified in three broad categories based on their clinical roles, viz., *doctors, nurses and other professional (includes: lab technicians, phlebotomist, dieticians, administration staff in hospital, clinical pharmacists).*

In the population, majority (1109, 55.2%) of the respondents were practising at hospitals having ICU facilities, and among them mostly were (738, 66.6%) were females. The remaining respondents (899, 44.8%) were practising at hospitals without having ICU facilities, among them majority were males (610, 67.9%). Most of females HCPs (71.86%)

were working in Hospital having ICU facilities, whereas, most of males HCPs (62.18%) were engaged in Hospital don not have ICU facilities. Mean years of experience in the field of HCPs were 11.0[± 15.8] years, females (mean 7.1 [± 18.7] years) were less experienced than males (mean 15.1 [± 10.4] years) (Table 1).

<<**Insert Table 1 about here**>>

Mean score on intrusion scale (intrusive thoughts related to trauma, flashbacks and recollections, a symptom of PTSD) was found to be 10.2 ±3.75); of which female reported a mean score of 10.7 (± 3.33), which is slightly higher than males (mean score of 9.62 (± 4.08)), which was indicating high intrusive thoughts among females than males.

On the avoidance scale (deals with the attempts to avoid any stimuli or triggers that might be related to the traumatic event). The participants reported an overall mean score of 14.4 (±4.6); mean score of females being 14.6 (±4.3) whereas males mean score being 14.2 (±4.9); indicating both males and female utilizing avoidance as a coping strategy. On the arousal scale (indicates jumpiness, irritability, insomnia, decreased concentration and hyper vigilance). Participants reported mean score of 10.8 (± 3.56); females reporting a mean score of 10.7 (± 3.4) and males mean score of 10.8 (± 3.7) which indicated psychosomatic arousal experienced by the participants due to their exposure with secondary trauma experienced by interaction with their patients. (Table 1)

Secondary traumatic stress

The key clinical characteristics of the present study are the STSS and LOTR levels of the HCPs. Secondary traumatic stress (STS) was experienced by 1548 (77%) of the HCPs and the remaining 23% were experienced either no STS or little STS in this pandemic situation, among them mostly (41.1%) were those were engaged in other professional activities. The severe STS level was found among doctors (116, 19.0%), which higher than that among nurses (76, 8.2%) and other HCPs (35, 7.4%). Similarly, high (74, 12.1%) and moderate STS level (129, 21.1%) was also found higher among doctors than that of among nurses and other HCPs. (Table 2)

<<**Insert Table 2 about here**>>

In comparison with the gender in “mild STS” male doctors (above 30%) were higher than female doctors; female nurses (above 40%) were higher than male nurses and other male HCPs (above 30%) were higher than other female HCPs. In “moderate STS” female doctors (above 20%) were higher than male doctors; male nurses (above 20%) were higher than female nurses and other female HCPs (above 20%) were higher than other male HCPs. In “high STS” male and female doctors were equal (above 10%); male and female nurses were equal (10%) and other male HCPs were higher than other female HCPs. In “severe STS” female doctors (above 20%) were higher than male doctors; male and female nurses were equal (10%) and other female HCPs (10%) were higher than other male HCPs. (Figure-1).

<<**Insert Figure 1 about here**>>

Optimism (LOT-R) and perceived Mood state.

Life Orientation Test-Revised (LOT-R) is another key component, which measures the dispositional optimism of an individual. Table 1 shows that among the HCPs, high Optimism or low pessimism was mostly observed among doctors (244, 39.3%), followed by nurses (247, 26.7%) and others professionals (108, 22.8%), whereas, in cases of low optimism or high pessimism category, the order changed i.e., other professionals (73, 15.4%), followed by doctors (67, 11.0%) and nurses (86, 9.3%). (Figure-2)

<<**Insert Figure 2 about here**>>

The perceived mood state of the HCPs was accessed with the help of a mood visual analogue scale (11 point Likert scale; where 0= extremely sad and 11 = extremely happy); the overall mean for the sample was found to be 5.68 (± 2.26) indicating moderate mood states reported by participants at the time of taking the survey. Gender-wise mean mood VAS score are quite closure to one another [females: 5.71 (± 2.34) and males: 5.65 (± 2.18)].

In mood VAS among females, nurses were higher on neutral mood than doctors and others. Nurses were higher on VAS between the points 6-10 indicating happiness compared to doctors and others. In mood VAS among males, others HCPs were higher on neutral mood

than doctors and nurses. Doctors were higher on VAS between the points 6-10 indicating happiness compared to nurses and others.

In comparison with the gender in doctors' males were higher on normal mood and higher on the points 6-10 indicating happiness compared to females; in nurses' females were higher on normal mood and higher on the points 6-10 indicating happiness compared to males and in in nurses' females were higher on normal mood and higher on the points 6-10 indicating happiness compared to males and in others males were higher on normal mood and higher on the points 6-10 indicating happiness compared to females. (Figure-3)

<<**Insert Figure 3 about here**>>

In regression analysis, doctors and nurses showed happier mood when compared to others HCPs. In STS, doctors and nurses showed more STS than others HCPs, and STS decreased with increase in the age. Regression model for the LOT-R, it was found that doctors and nurses were shown higher optimism than others HCPs. Regression model for the mood VAS, it observed that females HCPs experiences higher sad mood as compared to males. (Table 3)

<<**Insert Table 3 about here**>>

6. Discussion

Any pandemics remain a great source of threat to all communities due to the sudden spread and lack of immediate measure to control them. COVID-19 came with a similar threat package; due to its highly contagious nature it has become a source of great fright in the current times, and the fear responses have been quite evident in the frontline health care providers as well. The current study showed that 77% (N=1548) of HCPs (doctors, nurses, other non-medical health care providers) reported prevalence of STS and only 23% (N=460) reported no or little secondary traumatic stress. Severe STS was highly reported among doctors as compared to nurses and others, which is similar to earlier study's findings published during the pandemic[28]. Results associated with STS also depicts that no STS level was high among male engaged in others professional works, than that of females, and

was also higher than that of doctors and nurses of both genders. On the other hand, doctors of both genders were higher in the categories high and severe categories of STS, than that of nurses and other professionals'.

There was a difference in the patterns of responses among female and male participants who showed signs of intrusive thoughts, using avoidance as a coping mechanism and arousal when faced by triggers in the environment. The results show that female health care professionals showed higher levels of secondary traumatic stress (also related to symptoms of post-traumatic stress) as compared to their male counterparts, especially doctors and nurses as compared to other health care professionals. In studies conducted in India related to burnout and distress among doctors and nurses during the time of COVID-19,the health care professionals also showed significant burnout due to their direct contact and involvement in their work with pandemic related work and patient involvement. [7,8] Though no comparative data exists for pre-COVID secondary traumatization studied in Indian health care professionals ; the symptoms of burnout and effects of professional life experienced could be doctors, residents, nurses have shown that the conditions have been difficult ever since. [24, 25, 26]

The conditions are difficult for those directly involved in dealing with the crisis in the times of epidemic [25]. A study by Li et al. informed that vicarious traumatization adversely affected both medical and non-medical staff; also the vicarious traumatization was worse in non-front line medical workers as compared by frontline medical staff[27].

The current study also shows light on the reported mood states along with the traumatic stress and pessimism experienced during the course of their work in the field as frontline workers. The results show that neutral to lower moods were recorded across spectrum between both male and female health care professionals. The findings are in line with the similar findings among healthcare professionals during the pandemic conditions in India; where they showed signs of various mood and anxiety disorder like symptoms [28, 29]

The STS and burnouts have been reported higher in other studies as well, where the data collection was during a similar period of COVID-19 spread peak [30]. The results have been consistent in studies showing health care providers response to working earlier in adverse conditions during SARS pandemic [31]. To prevent severe psychological distress and

negative mental health outcomes; it is the need of the hour to provide better mental health care facilities for the health care providers.

The need for immediate intervention also reflects in the low optimism reported by all healthcare providers in the study. The lower optimism could have been due to various factors such as lesser years of experience in the field, and no exposure to working in adverse conditions with lack of critical medical supplies. Age-related variance in the study could have also played an essential role in contributing to optimism along with marital status contributing to fear of transmission to family members, loss of job [32].

As the spread of the pandemic takes a sharp hike in the Indian subcontinent, the disease related psychological burden also spirals in the neglected healthcare providers. Identifying and addressing these mental health issues and ensuring both physical and psychological safety should become the priority for not only front liners but for everyone in the field.
LOT-R among females based on the professional categories shows that those engaged in others professional works mostly belongs to “moderate-optimism” and high optimism categories; among nurses mostly belongs to “moderate-optimism” category; doctors mostly belongs to “moderate to severe optimism” categories. LOT-R among males shows that those engaged in others professional works most of them belongs to each of “moderate-optimism” category; among nurses mostly belongs to each of “moderate and high-optimism” categories; doctors also mostly belongs to “moderate and high-optimism” categories like nurses.
Increase in pessimistic view for outcome of future could be an indicator for the healthcare professionals and can predict negative effects of engagement with the pandemic hit population on their personal psychological wellbeing as well. [33]

7. Limitations

Irrespective of the large data set and strength of the study, there are certain limitations. First, is the utilization of the cross-sectional design, lack of homogeneity at various levels, and over-representation of a particular group of healthcare providers could have played a mediating role in the results; which also would interfere with causality analysis between the variables of the study. Second, the participants recruited with the help of social media forums, along with snowballing method; which again dictated the overrepresentation of a

specific group of practitioners; who first all accessed social media regularly and had access to internet facilities.

More than that, psychological health has been proven to be influenced by various factors, secondary traumatic stress could be influenced not only by the current pandemic but other factors as well in the healthcare providers. Though COVID-19 has increased the workload and safety concerns, other factors such as family support, job stress, disturbed daily activities could have contributed significantly to the overall health and quality of life.

8. Conclusion

~~Despite certain limitations, the current study was able to explore the role of HCPs expanding beyond their areas of specialization due to lack of availability of trained/ expert professionals to keep up with the increasing demand.~~ This could have been an important intervening factor to create distress, burnout and make the HCPs vulnerable to various mental health and physical health issues during the pandemic.

The immediate need for focus on the secondary traumatic stress experienced by the healthcare provider, and related burden it brings along—the decline in optimistic thinking to improve the work-life balance and dealing with work-related stressors. Further, emphasizing the need for social and administration level support in helping build better healthcare policies and mental health support system.

Ethics statement

The approval was obtained from The Ethics committee of Manipal hospitals, Bangalore (ECR/34/Inst/KA/2013/RR-19). Our investigation process remained anonymous, and no identifiers (such as name, address, email id, phone numbers, name of hospital employed) were collected. Every participant was informed about and understood the purpose of our investigation before entering the study.

References

1 .Greinacher A., Derezza-Greeven C., Herzog W., Nikendei C. Secondary traumatization in first responders: A system-atic review. *Eur. J. Psychotraumatology.* 2019;**10**:1562840.

2. Conversano C., Marchi L., Miniati M. Perspective article Psychological distress among healthcare professionals in-volved in the Covid-19 emergency: Vulnerability and resilience factors CiroConversano, Laura Marchi, Mario Miniati. *Clin. Neuropsychiatry.* 2020;**17**:94–96.

3. Zhang W.-R., Wang K., Yin L., Zhao W.-F., Xue Q., Peng M., Min B.-Q., Tian Q., Leng H.-X., Du J.-L., et al. Mental Health and Psychosocial Problems of Medical Health Workers during the COVID-19 Epidemic in China. *Psychother. Psychosom.* 2020;**89**:242–250.

4. Chen Q., Liang M., Li Y., Guo J., Fei D., Wang L., He L., Sheng C., Cai Y., Li X., et al. Mental health care for medical staff in China during the COVID-19 outbreak. *Lancet Psychiatry.* 2020;**7**:e15–e16.

5. Lu W., Wang H., Lin Y., Li L. Psychological status of medical workforce during the COVID-19 pandemic: A cross-sectional study. *Psychiatry Res.* 2020;**288**:112936.

6. Pappa S., Ntella V., Giannakas T., Giannakoulis V.G., Papoutsi E., Katsaounou P. Prevalence of depression, anxiety, and insomnia among healthcare workers during the COVID-19 pandemic: A systematic review and meta-analysis. *Brain Behav. Immun.* 2020;**88**:901–907.

7. Khasne, R. W., Dhakulkar, B. S., Mahajan, H. C., & Kulkarni, A. P. (2020). Burnout among Healthcare Workers during COVID-19 Pandemic in India: Results of a Questionnaire-based Survey. *Indian journal of critical care medicine : peer-reviewed, official publication of Indian Society of Critical Care Medicine*, *24*(8), 664–671.

8. Jose S, Dhandapani M, Cyriac MC. Burnout and Resilience among Frontline Nurses during COVID-19 Pandemic: A Cross-sectional Study in the Emergency Department of a Tertiary Care Center, North India. Indian J Crit Care Med. 2020;24(11):1081-1088.

9. Figley CR, Carbonell JL, Boscarino JA, Chang J. A clinical demonstration model for assessing the effectiveness of therapeutic interventions: an expanded clinical trials methodology. *Int J EmergMent Health.* (1999) 1:155–64.

10. American Psychiatric Association *Diagnostic and Statistical Manual of Mental Disorders, 4th Edn, Text Revision.* Washington, DC: American Psychiatric Publishing; (2000).

11. Bride BE, Robinson MM, Yegidis B, Figley CR. Development and validation of the secondary traumatic stress scale. *Res Soc Work Pract.* (2004) 14:27–35.

12. Neto, M. L., Almeida, H. G., Esmeraldo, J. D., Nobre, C. B., Pinheiro, W. R., Oliveira, C. R., Silva, C. G. When health professionals look death in the eye: The mental health of professionals who deal daily with the 2019 coronavirus outbreak. Psychiatry Research, 2020;288, 112972.

13. COVID-19 outbreak: Mental health professionals raise alarm. (2020, June 5). Retrieved from https://www.timesnownews.com/health/article/covid-19-outbreak-mental-health-professionals-raise-alarm/601852

14. Tsamakis K, Rizos E, Manolis AJ, Chaidou S, Kympouropoulos S, Spartalis E, Spandidos DA, Tsiptsios D, Triantafyllis AS. COVID-19 pandemic and its impact on mental health of healthcare professionals. ExpTher Med. 2020;19(6):3451-3453.

15. Yildirim, M., Arslan, G., &Özaslan, A. Perceived Risk and Mental Health Problems Among Healthcare Professionals During COVID-19 Pandemic: Exploring the Mediating Effects of Resilience and Coronavirus Fear. 2020;

16. Lai J., Ma S., Ma, Wang Y., Cai Z., Hu J., Wei N., Wu J., Du H., Chen T., Li R., Tan H., Kang L., Yao L., Huang M., Wang H., Wang G., Liu Z., Hu S. Factors associated with mental health outcomes among health care workers exposed to coronavirus disease. JAMA Network Open. 2020

17. Boldor N, Bar-Dayan Y, Rosenbloom T, Shemer J, Bar-Dayan Y. Optimism of health care workers during a disaster: a review of the literature. Emerg Health Threats J. 2012;5.

18. Sim MR. The COVID-19 pandemic: major risks to healthcare and other workers on the front line. Occup Environ Med. 2020;77(5):281-282.

19. Arpacioglu S, Gurler M, Cakiroglu S. Secondary Traumatization Outcomes and Associated Factors Among the Health Care Workers Exposed to the COVID-19. Int J Soc Psychiatry. 2020 (8):20764020940742

20. Bride, B. E., Hatcher, S. S., & Humble, M. N. (2009). Trauma training, trauma practices, and secondary traumatic stress among substance abuse counselors. *Traumatology, 15*(2), 96–105.

21. Elwood LS, Mott J, Lohr JM, Galovski TE. Secondary trauma symptoms in clinicians: a critical review of the construct, specificity, and implications for trauma-focused treatment. ClinPsychol Rev. 2011 Feb;31(1):25-36.

22. J. Gustems-Carnicer, C. Calderón, M. Forn. Psychometric properties of the Life Orientation Test (LOT-R) and its relationship with psychological well-being and academic progress in college students.RevistaLatinoamericanadePsicología. (2016).

23. Flynn D, van Schaik P, van Wersch A. A comparison of multi-item likert and visual analogue scales for the assessment of transactionally defined coping. Eur J Psychol Assess. 2004;20:49–58.

24. Grover S, Sahoo S, Bhalla A, Avasthi A. Psychological problems and burnout among medical professionals of a tertiary care hospital of North India: A cross-sectional study. Indian JPsychiatry.2018 Apr-Jun;60(2):175-188.

25. Saravanabavan, L., Sivakumar, M. N., & Hisham, M. (2019). Stress and Burnout among Intensive Care Unit Healthcare Professionals in an Indian Tertiary Care Hospital. *Indian journal of critical care medicine : peer-reviewed, official publication of Indian Society of Critical Care Medicine*, *23*(10), 462–466.

26. Magnavita N, Soave PM, Ricciardi W, Antonelli M. Occupational Stress and Mental Health among Anesthetists during the COVID-19 Pandemic. Int J Environ Res Public Health. 2020 Nov 8;17(21):8245.

27. Li Z, Ge J, Yang M, Feng J, Qiao M, Jiang R, Bi J, Zhan G, Xu X, Wang L, Zhou Q, Zhou C, Pan Y, Liu S, Zhang H, Yang J, Zhu B, Hu Y, Hashimoto K, Jia Y, Wang H, Wang R, Liu C, Yang C. Vicarious traumatization in the general public, members, and non-members of medical teams aiding in COVID-19 control. Brain Behav Immun. 2020;88:916-919.

28. Folstein MF, Luria R. Reliability, validity, and clinical application of the visual analogue mood scale. Psychological Medicine. Cambridge University Press; 1973;3(4):479–86.

29. Suryavanshi, N, Kadam, A, Dhumal, G, et al. Mental health and quality of life among healthcare professionals during the COVID-19 pandemic in India. *Brain Behav*. 2020; 10:e01837. https://doi.org/10.1002/brb3.1837

30. Kannampallil TG, Goss CW, Evanoff BA, Strickland JR, McAlister RP, et al. (2020) Exposure to COVID-19 patients increases physician trainee stress and burnout. PLOS ONE 15(8):

31. Zhang L. J., Lu P. J., & Gao Y. X. (2004). A study on the emotional and behavioral experiences of nursing students during the SARS epidemic. Journal of Nurses Training, 19(7): 601–603.

32. Matheson C, Robertson HD, Elliott AM, Iversen L, Murchie P. Resilience of primary healthcare professionals working in challenging environments: a focus group study. Br J Gen Pract. 2016;66(648):e507–e515. pmid:27162205

33. Arslan, G., Yıldırım, M., Tanhan, A. *et al.* Coronavirus Stress, Optimism-Pessimism, Psychological Inflexibility, and Psychological Health: Psychometric Properties of the Coronavirus Stress Measure. *Int J Ment Health Addiction* .

 Click here to access/download;Figure;0 Figure.docx

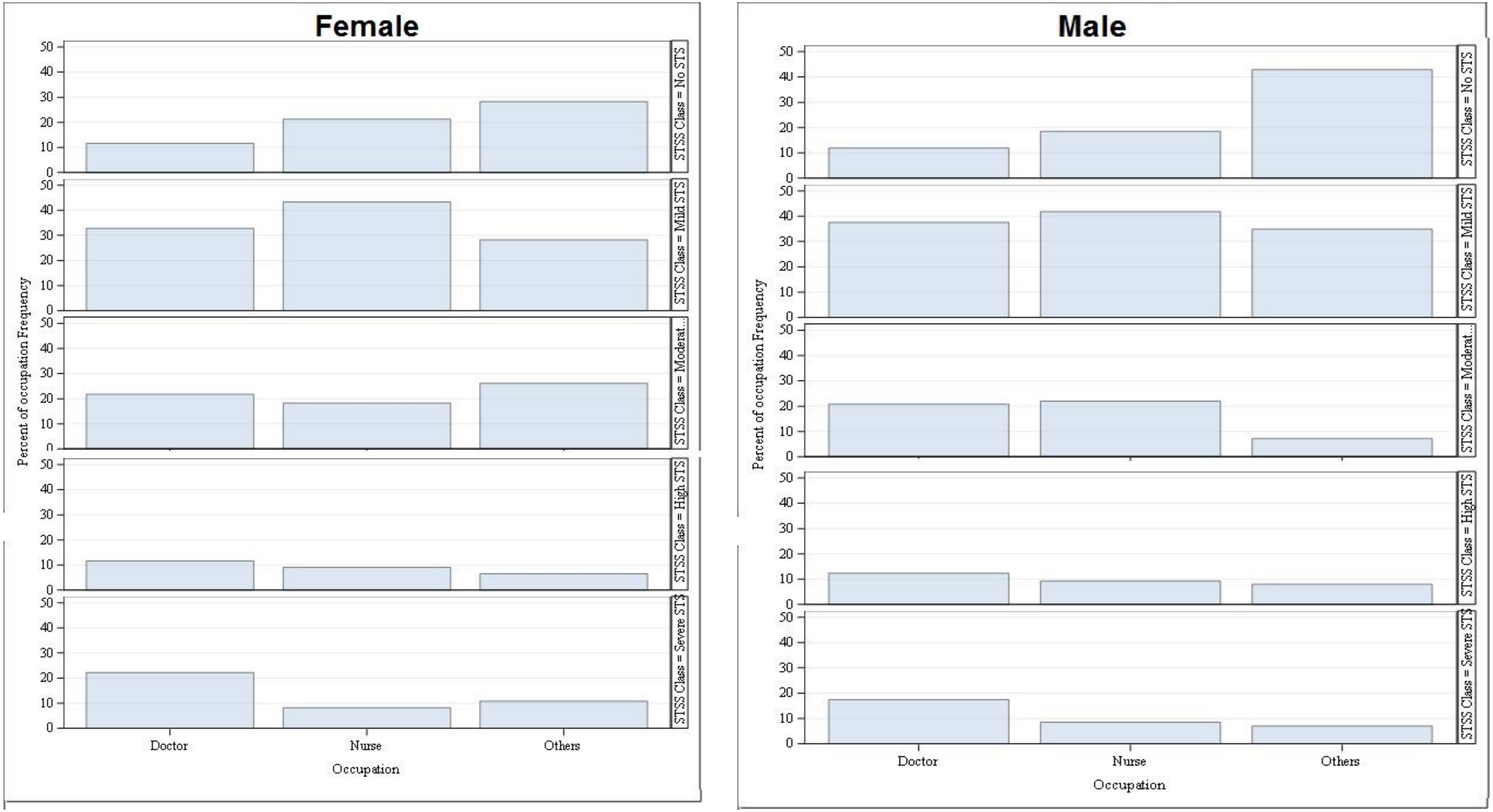

Figure 1: Occupation-wise distribution of severity of secondary traumatic stress of HCPs based on their gender.

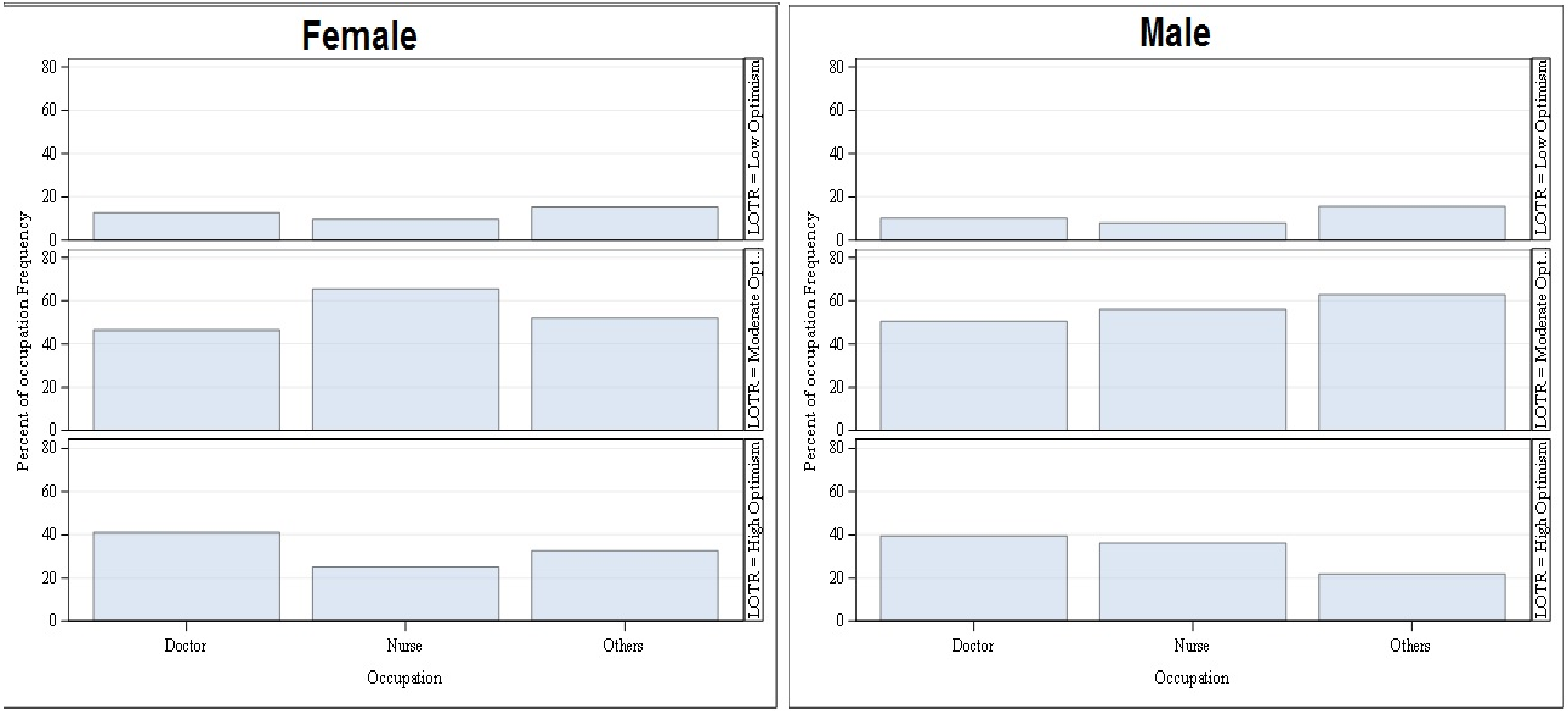

.

Figure 2: Occupation-wise distribution of varying levels of optimism/pessimismusing Life Orientation Test-Revised of HCPs based on their gender

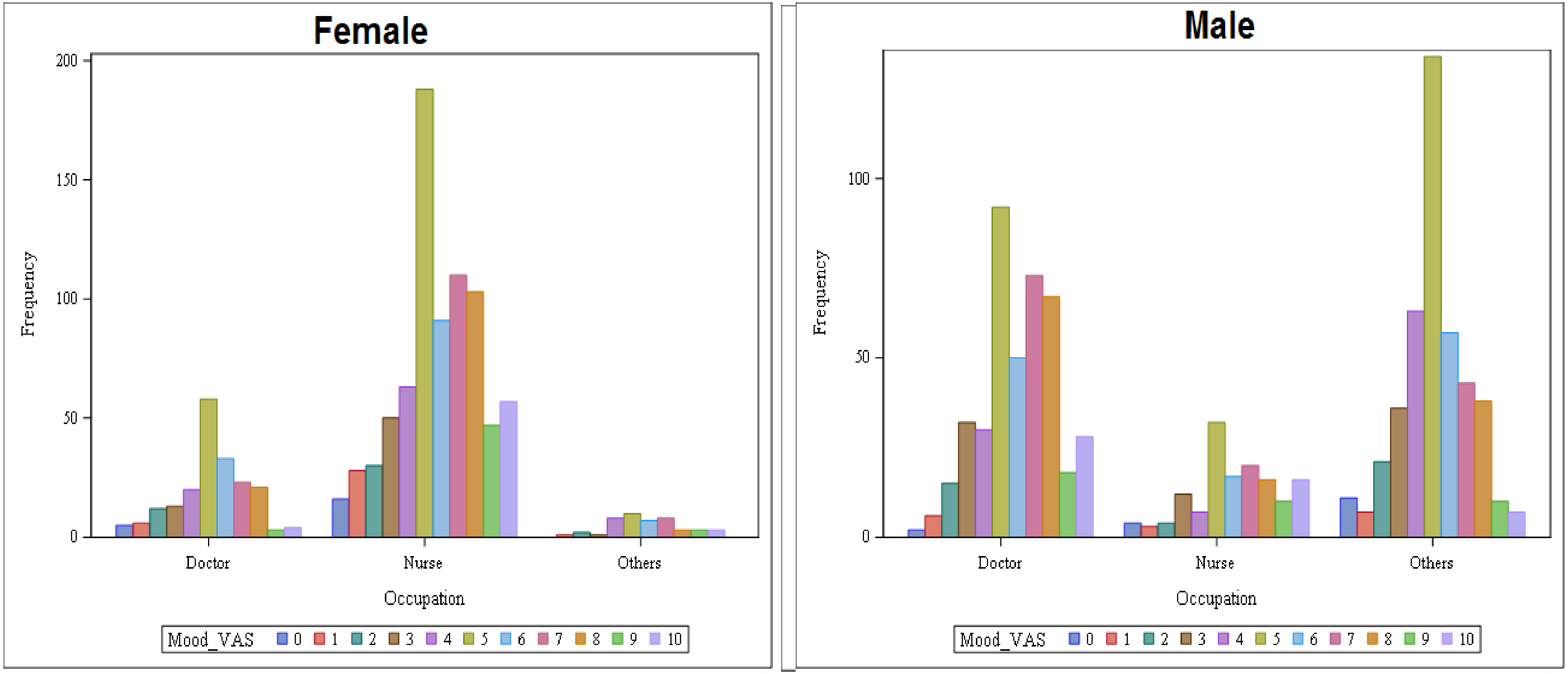

Figure 3: Occupation-wise distribution of mood status of HCPs based on their gender.

Tables Click here to access/download;Figure;0 Table.docx

Table 1. Mean and standard deviations of the scores obtained on socio-demographic details along with overall secondary traumatic stress (STS) and Mood visual analog scale responses.

**Name of Characteristics**	**Total**		**Female**		**Male**		**p-value**
	N	%	N	%	N	%	
Age mean (SD)	35.7 (11.9)		29.7 (8.9)		41.9 (11.5)		
Marital status							
• Married	1208	60.2	421	41.0	787	80.2	<.0001
• Unmarried	800	39.8	606	59.0	194	19.8	<.0001
Clinical role							
• Doctor	611	30.4	198	19.3	413	42.1	<.0001
• Nurse	924	46.0	783	76.2	141	14.4	<.0001
• Others	473	23.6	46	4.5	427	43.5	<.0001
Type of practice							
• Hospital without ICU	899	44.8	289	28.1	610	62.2	<.0001
• Hospital with ICU	1109	55.2	738	17.9	371	37.8	<.0001
Experience in the field (years) (Mean (SD))	11.0 (15.8)		7.1 (18.7)		15.1 (10.4)		<.0001
Intrusion (Mean (SD))	10.2(3.8)		10.7(3.3)		9.6(4.1)		<.0001
Avoidance(Mean(SD))	14.4(4.6)		14.6(4.3)		14.2(4.9)		<.0001
Arousal(Mean (SD))	10.8(3.6)		10.7(3.4)		10.8(3.7)		<.0001
Mood VAS (Mean (SD))	5.7(2.3)		5.7(2.3)		5.7(2.2)		<.0001

Table 2. Descriptive data including frequency and percentage for the health care professionals and severity of secondary traumatic stress and varying levels of optimism/pessimismusing Life Orientation Test-Revised
.

Variable	Occupation				p-value
	Total (2008)	Doctor (n=611)	Nurse (n=924)	Others (n=473)	
**STSS**					<.0001
• No STS	460	72 (11.8)	192 (20.8)	196 (41.4)	
• Mild STS	780	220 (36.0)	398 (43.1)	162 (34.3)	
• Moderate STS	346	129 (21.1)	174 (18.8)	43 (9.1)	
• High STS	195	74 (12.1)	84 (9.1)	37 (7.8)	
• Severe STS	227	116 (19.0)	76 (8.2)	35 (7.4)	
**Intrusion (mean(SD))**		11.7(3.7)	10.4(3.1)	7.8(3.9)	<.0001
**Avoidance(mean(SD))**		15.8(4.8)	14.4(4.0)	12.7(4.8)	<.0001
**Arousal(mean(SD))**		11.8(3.7)	10.3(3.2)	10.2(3.8)	<.0001
**LOTR**					<.0001
• Low Optimism (High pessimism)	226	67 (11.0)	86 (9.3)	73 (15.4)	
• Moderate Optimism	1183	300 (49.1)	591 (64.0)	292 (61.7)	
• High Optimism (Low pessimism)	599	244 (39.3)	247 (26.7)	108 (22.8)	
**Mood VAS**		5.8(2.2)	5.9(2.4)	5.2(2.0)	<.0001

Table 3: Details of Regression model for STSS, LOTR and Mood VAS and its associated covariates selected through stepwise procedure.

Parameter		Estimates	Standard Error	Pr > |t|
**STSS**				
**Intercept**		35.74231	1.134083	<.0001
**Age**		-0.1168	0.023984	<.0001
**Occupation**				
	Doctor	8.496866	0.611651	<.0001
	Nurse	2.556519	0.678363	0.0002
	Others^R			
**LOTR**				
**Intercept**		2.073996	0.027992	<.0001
**Occupation**				
	Doctor	0.215693	0.037284	<.0001
	Nurse	0.100247	0.034419	0.0036
	Others^R			
**Mood_VAS**				
**Intercept**		5.226624	0.103963	<.0001
**Gender**				
	Female	-0.37377	0.13069	0.0043
	Male^R			
**Occupation**				
	Doctor	0.678459	0.140597	<.0001
	Nurse	0.953744	0.160338	<.0001
	Others^R			
