# Supplementary material for: Prevalence and severity of secondary traumatic stress and optimism in Indian health care professionals during COVID-19 lockdown
Source: PLoS One. 2021 Sep 28;16(9):e0257429. doi: 10.1371/journal.pone.0257429 (PMC8478227; doi:10.1371/journal.pone.0257429)
Supplement: Attachment — Submitted filename: RESPONSE TO REVIEWERSdocx.docx [file pone.0257429.s003.docx]

**RESPONSE TO REVIEWERS’REPORT(S)**

| **Reviewer 1** | **Response** |
| --- | --- |
| (a) Since Doctors and Nurses reported to have high level of stress, more among doctors-author can a make statement about the relationship of specialty of a doctor and experienced stress. | During the COVID-19 lockdown period, all the doctors irrespective of their specialization were involved in COVID duties only. Based on their role in broader perspective the clinical group is classified into three subgroups.  [Page-5, paragraph 2; line 5-8] |
| (b) Author can share recommendations about management of stress for HCPs, it will be helpful for policy makers to implement. | Page-13, paragraph 4; line 5-10 |
| (c) Author can specify the Cronbach alpha of the used tests for the study. | 1. Secondary traumatic Stress Scale (Page-6, paragraph 2)  2. Life Orientation Test-Revised (Page-6, paragraph 3)  3. Mood (VAS) (Page-7, paragraph 1)  *New reference added for Mood (VAS) Cronbach score.*  23. Flynn D, van Schaik P, van Wersch A. A comparison of multi-item likert and visual analogue scales for the assessment of transactionally defined coping. Eur J Psychol Assess. 2004;20:49–58. |
| **Reviewer 2** | **Response** |
| There are definitely few grammatical errors which must be addressed. | Needful changes have been incorporated at various places in the revised manuscript. (*yellow highlights, apart from the changes mentioned in the list).* |
| Authors need to mention more Indian studies - few are from Indian Journal of Psychiatry and Asian Journal of Psychiatry. | Page-12, paragraph 2 and Page-14, paragraph 4; line 4-10 (multiple new and relevant Indian studies added).  33. Arslan, G., Yıldırım, M., Tanhan, A. *et al.* Coronavirus Stress, Optimism-Pessimism, Psychological Inflexibility, and Psychological Health: Psychometric Properties of the Coronavirus Stress Measure. *Int J Ment Health Addiction* .  34. Mathur S, Sharma D, Solanki RK, Goyal MK. Stress-related disorders in health-care workers in COVID-19 pandemic: A cross-sectional study from India. Indian J Med Spec 2020;11:180-4  35. Chatterjee SS, Chakrabarty M, Banerjee D, Grover S, Chatterjee SS, Dan U. Stress, Sleep and Psychological Impact in Healthcare Workers During the Early Phase of COVID-19 in India: A Factor Analysis. Front Psychol. 2021 Feb 25;12:611314.  36. Singh RK, Bajpai R, Kaswan P. COVID-19 pandemic and psychological wellbeing among health care workers and general population: A systematic-review and meta-analysis of the current evidence from India. Clin Epidemiol Glob Health. 2021 Jul-Sep;11:100737.  37. Grover S, Dua D, Shouan A, Nehra R, Avasthi A. Perceived stress and barriers to seeking help from mental health professionals among trainee doctors at a tertiary care centre in North India. Asian J Psychiatr 2019;39:143-9.  38. Banerjee D, Vijayakumar HG, Rao T S. ”Watching the watchmen:” Mental health needs and solutions for the health-care workers during the coronavirus disease 2019 pandemic. Int J Health Allied Sci 2020;9, Suppl S1:51-4 |
| **Other comments/ suggestions:**  1. Change in the name from Diagnostic and Statistical Manual of Psychiatric Disorders and DSM-IV”.  2. Mention the timeline of first national lockdown. | **1. Page-3, paragraph 43 lines 6** to “Diagnostic and Statistical Manual of Mental Disorders and DSM-IV-TR”.)  **2. Page-5, paragraph 1, line 8** (The first lockdown was initially stated to be from 25 March 2020 – 14 April 2020 but it was extended post April 14.-in certain states.) |
| 1.Change in table numbering (as table number 1 related to STSS score was added). | **1. Table 1 :** Page 6 (new addition)  **2. Earlier Table 1= Current Table 2** (page-8)  3. **Earlier Table 2= Current Table 3** (page-10)  4. **Earlier Table 3= Current Table 4** (page-11)  *****Table numbers were also updated respectively in the revised manuscript (wherever mentioned). |

**Note: All of the changes in the revised manuscript are highlighted using YELLOWcolour.**
